# Interplay among ATP-Dependent Chromatin Remodelers Determines Chromatin Organisation in Yeast

**DOI:** 10.3390/biology9080190

**Published:** 2020-07-25

**Authors:** Hemant K. Prajapati, Josefina Ocampo, David J. Clark

**Affiliations:** 1 Division of Developmental Biology, NICHD, National Institutes of Health, Bethesda, MD 20892, USA; hemant.prajapati@nih.gov; 2 Instituto de Investigaciones en Ingeniería Genética y Biología Molecular “Dr. Héctor N. Torres” (INGEBI-CONICET), Buenos Aires C1428ADN, Argentina

**Keywords:** Chromatin, Chromatin remodelers, ISW1, ISW2, INO80, CHD1, SWI/SNF, RSC, nucleosome spacing, nucleosome phasing

## Abstract

Cellular DNA is packaged into chromatin, which is composed of regularly-spaced nucleosomes with occasional gaps corresponding to active regulatory elements, such as promoters and enhancers, called nucleosome-depleted regions (NDRs). This chromatin organisation is primarily determined by the activities of a set of ATP-dependent remodeling enzymes that are capable of moving nucleosomes along DNA, or of evicting nucleosomes altogether. In yeast, the nucleosome-spacing enzymes are ISW1 (Imitation SWitch protein 1), Chromodomain-Helicase-DNA-binding (CHD)1, ISW2 (Imitation SWitch protein 2) and INOsitol-requiring 80 (INO80); the nucleosome eviction enzymes are the SWItching/Sucrose Non-Fermenting (SWI/SNF) family, the Remodeling the Structure of Chromatin (RSC) complexes and INO80. We discuss the contributions of each set of enzymes to chromatin organisation. ISW1 and CHD1 are the major spacing enzymes; loss of both enzymes results in major chromatin disruption, partly due to the appearance of close-packed di-nucleosomes. ISW1 and CHD1 compete to set nucleosome spacing on most genes. ISW1 is dominant, setting wild type spacing, whereas CHD1 sets short spacing and may dominate on highly-transcribed genes. We propose that the competing remodelers regulate spacing, which in turn controls the binding of linker histone (H1) and therefore the degree of chromatin folding. Thus, genes with long spacing bind more H1, resulting in increased chromatin compaction. RSC, SWI/SNF and INO80 are involved in NDR formation, either directly by nucleosome eviction or repositioning, or indirectly by affecting the size of the complex that resides in the NDR. The nature of this complex is controversial: some suggest that it is a RSC-bound “fragile nucleosome”, whereas we propose that it is a non-histone transcription complex. In either case, this complex appears to serve as a barrier to nucleosome formation, resulting in the formation of phased nucleosomal arrays on both sides.

## 1. Introduction

The nucleosome core contains about 147 bp of DNA coiled about 1.7 times around a central octamer of core histones comprised of two molecules of each core histone (H3, H4, H2A and H2B), organised as a central (H3–H4)_2_ tetramer flanked by H2A–H2B dimers [1]. This structure is very compact, limiting access to the DNA. Consequently, nucleosomes generally inhibit DNA-dependent processes such as transcription and replication in vitro. It is thought that cells exploit the intrinsically-inhibitory properties of the nucleosome to regulate gene expression and cellular differentiation. For example, the nucleosome may block access to promoter or enhancer DNA, thereby preventing inappropriate gene activation. It follows that the cell needs to regulate chromatin structure in order to activate or repress specific genes in specific cell types. Accordingly, cells possess many enzymes and proteins that coordinate this regulation, which can be divided into three broad classes: (i) ATP-dependent chromatin remodeling enzymes which manipulate nucleosome occupancy, structure and position; (ii) histone-modification enzymes which catalyse, recognise or remove post-translational modifications of the histones (the histone code hypothesis [2]) and (iii) histone chaperones, which are required for histone transfer to and from DNA. Here, we discuss the ATP-dependent chromatin remodeling enzymes with a focus on the budding yeast enzymes, which are probably the best-studied both in vitro and in vivo. We note that most of them are conserved in higher organisms [3,4], and that mutations in many remodelers have been strongly linked to various cancers [5].

**Figure 1:**
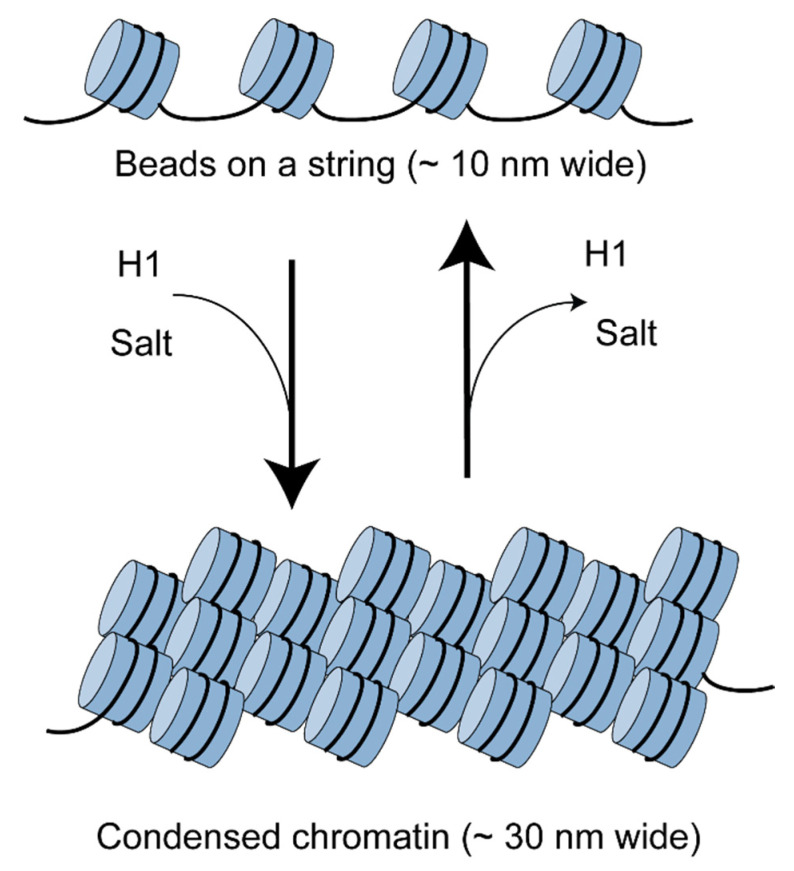
Chromatin is composed of regularly-spaced nucleosomes. This beads-on-a-string structure is visible in the electron microscope at very low salt concentrations. At physiological salt concentration, chromatin folds to form a condensed fibre about 30 nm wide, facilitated by the linker histone (H1).

Some of the earliest electron microscopy and nuclease digestion studies in the chromatin field revealed that nucleosomes are regularly spaced in chromatin, like beads on a string [6]. Each bead represents a nucleosome core; the intervening string is the linker DNA, connecting one nucleosome core to the next (Figure 1). The linker histone, H1, binds to the DNA entry–exit point in the nucleosome core and to the linker DNA, directing the condensation of the beads-on-a-string structure to form a somewhat irregular fibre about 30 nm wide [6,7]. It is worth noting that the beads-on-a-string organisation is only clearly visible at salt concentrations far below physiological, when the chromatin fibre expands due to electrostatic repulsions between linkers [7,8]. At salt and magnesium concentrations in the physiological range, the beads-on-a-string fibre spontaneously condenses to form the 30 nm fibre, most likely involving helical coiling [6,9]. The precise conformation of the folded chromatin fibre has been hotly debated for decades with no real consensus, mostly because the structure is not uniform and each technique employed to discern its structure has its own advantages and drawbacks.

The underlying beads-on-a-string structure is also apparent in nuclease digestion studies of nuclei, which reveal a “ladder” of DNA bands corresponding to multiples of a fixed distance between nucleosome cores, termed the nucleosome “repeat length” (equal to the nucleosome core plus the linker length) (reviewed in [10]) This pattern results from relatively-rapid digestion of the linker DNA, because DNA in the nucleosome core is protected from digestion by the core histones. The pattern indicates that most of the genome is organised into regularly-spaced nucleosomes. Regular spacing is observed in all eukaryotic cells from yeast to human, with some interesting variation. For example, budding yeast, fission yeast and some neurons have relatively short repeat lengths (~165 bp) and low H1 levels [11,12], whereas most cells in higher organisms have a longer repeat length (180–195 bp), and a few transcriptionally-inactive genomes have very long repeat lengths and special H1 variants (>200 bp). The repeat length is a global average; there is significant variation in linker length within cell populations, which is governed by the ‘10*n* + 5 bp’ rule (where *n* ≥ 0, such that linker lengths are 5, 15, 25 bp etc.) [13,14]. The biological significance of cell type-specific variation in nucleosome spacing is still unclear.

Nucleosomes can be reconstituted in vitro using only purified core histones and DNA, under special conditions to prevent aggregation and precipitation. Although such nucleosomes appear indistinguishable from those formed in cells, they do not form regularly-spaced arrays, i.e., the regular beads-on-a-string structure is absent. Instead, nucleosome formation is directed by DNA sequence preference (strongly influenced by sequence-dependent DNA bendability because nucleosomal DNA is highly bent), resulting in variable spacing. Nucleosomes are generally very stable structures at physiological ionic strength. Consequently, in vitro, regular spacing of reconstituted nucleosomes requires the addition of an ATP-dependent nucleosome-spacing enzyme, which harnesses the free energy of ATP hydrolysis to slide the nucleosomes into a regular array [15,16].

## 2. The ATP-Dependent Chromatin Remodelers

**Figure 2:**
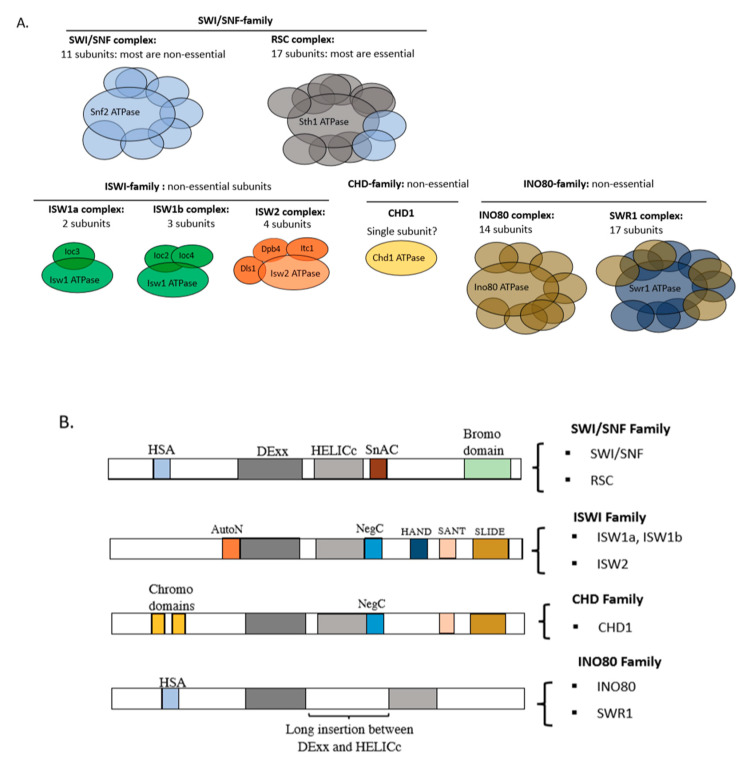
ATP-dependent chromatin remodelers in yeast. (**A**) Subunit organisation of remodeler families. All of the remodelers have a core ATPase subunit. A few subunits are found in more than one complex within a family (indicated by the same colour). (**B**) Remodelers are divided into four major families based on the combination of domains present in their catalytic subunits. The conserved ATPase domain has two lobes, DExx and HELICc, flanked by other domains, such as the AutoN and NegC domains in ISWI(Imitation SWItch)-enzymes, which are required for negative regulation of ATPase function, and the SnAc domain in the switching/sucrose non-fermenting (SWI/SNF) family (Snf2 ATPase coupling). Bromodomains and chromodomains recognise modified histones (see text). Adapted from [17].

Most ATP-dependent remodelers are multi-subunit complexes containing an ATPase subunit which catalyses remodeling (Figure 2A). The catalytic subunit generally has a well-conserved ATPase domain together with various flanking domains which divide the remodelers into four major families, defined by the SWI/SNF, ISWI, CHD and INO80 complexes [17]. The ATPase domain contains RecA-like lobes (DExx and HELICc) which are conserved in all four families (Figure 2B). The SWI/SNF (switching/sucrose non-fermenting) and RSC (remodeling the structure of chromatin) complexes are the key members of the SWI/SNF family in budding yeast. Their ATPase subunits (Snf2 in SWI/SNF and Sth1 in RSC) have an N-terminal helicase-SANT (HSA) domain and a C-terminal bromodomain. The HSA domain binds the actin or actin-related protein (ARP) subunits, whereas bromodomains commonly bind acetylated histone residues and may be involved in targeting. Yeast SWI/SNF and RSC are very large complexes, containing 11 and 17 subunits, respectively. Remodelers in the ISWI (imitation switch) family tend to have fewer subunits. The ISWI family ATPase has a conserved C-terminal HAND-SANT-SLIDE (HSS) domain, which binds nucleosomes and DNA [17]. In yeast, there are three ISWI-family complexes with just two or three subunits—ISW1a, ISW1b and ISW2 (Figure 2A). The only CHD (chromodomain-helicase-DNA-binding) family remodeler in yeast, CHD1, consists of just an ATPase subunit. CHD1 has two tandem chromodomains at the N-terminus and a SANT-SLIDE domain at the C-terminus [18]. Many chromodomains recognise methylated histone residues [19,20], but this has not been demonstrated for CHD1. Finally, the INO80 (inositol-requiring 80) family ATPase subunit has a unique long insertion between the DExx and HELICc lobes (a “split” ATPase domain) and an N-terminal HSA domain that is similar to that of the SWI/SNF family [17,21,22]. The yeast representatives of the INO80 family are INO80 (14 subunits) and SWR1 (17 subunits).

The ATP-dependent chromatin remodeling enzymes can also be subdivided into three classes based on their activities: (1) Nucleosome-spacing enzymes which slide nucleosomes along DNA to fix their spacing (i.e., the linker length). In yeast, these are CHD1, ISW1a, ISW1b, ISW2 and INO80. (2) Nucleosome-remodeling enzymes capable of sliding nucleosomes along DNA, inducing conformational changes in nucleosomes, removing nucleosomes from DNA or transferring nucleosomes from one DNA molecule to another [22]. In yeast, these enzymes are represented by the SWI/SNF and RSC complexes. (3) Histone-variant-exchange enzymes, such as the SWR complex, which uses ATP to replace an H2A–H2B dimer in a nucleosome with an H2A.Z-H2B dimer [23,24]. INO80 is exceptional in that it may display all three activities, although its role in exchange of H2A.Z-H2B dimers is controversial [25,26,27,28].

Relatively little is known about the roles of the ancillary subunits, given that the ATPase subunit contains the remodeling activity. These subunits may have regulatory functions, such as targeting a remodeler to specific DNA sequences (e.g., the Rsc3 and Rsc30 subunits of RSC [29]), or to nucleosomes carrying specific histone modifications (e.g., RSC has many bromodomains [30,31,32]).

## 3. Nucleosome Mapping: MNase-Seq and Chemical Mapping

The major approach employed to determine the contributions of the various ATP-dependent chromatin remodelers to chromatin organisation in cells is to map nucleosomes in a remodeler mutant and compare with wild type. Nearly all such studies have employed MNase-seq. This technique involves digestion of nuclei with micrococcal nuclease (MNase) to yield predominantly mono-nucleosomes (i.e., isolated beads from the beads-on-a-string), followed by purification of nucleosomal DNA, preparation of sequencing libraries and paired-end sequencing [33]. Paired-end sequencing yields short sequence reads from both ends of the same nucleosomal DNA molecule, which are then aligned to the genome. The length of the nucleosomal DNA fragment can be deduced from the distance between the 5′-ends of the two reads. The central base pair in the nucleosomal DNA molecule is assumed to represent the centre of the nucleosome, termed the dyad (in reference to the crystal structure) and is used to indicate the position of the nucleosome with respect to the genome. Nucleosome occupancy is represented by the coverage plot, which involves counting the number of times each chromosomal coordinate base pair occurs within a nucleosome sequence and normalising to the genomic average count (usually set at 1).

The expected length of the nucleosomal DNA is, of course, ~147 bp. In practice, a range of nucleosomal DNA fragment lengths is observed. DNA fragments that are too long may reflect slower digestion of linker DNA with higher GC content (MNase prefers AT-rich DNA), such that some linker DNA is still projecting out of the nucleosome, or possibly protection of linker DNA by other proteins. DNA fragments which are too short may result from over-digestion (digestion within the nucleosome at AT-rich sites) or perhaps to sub-nucleosomes (hexasomes and perhaps (H3–H4)_2_ tetrasomes), although these are difficult to distinguish from over-digestion because the same fragment sizes are produced in both cases. The marked preference of MNase for AT-rich DNA results in different digestion rates for linkers and nucleosomes, depending on their base composition, which can be an issue if delving deep into the data [34]. In our experience, the most critical parameter for obtaining a high quality nucleosome map is the extent of digestion. We always do an MNase titration and choose samples that are almost all mono-nucleosomes with minimal sub-nucleosomal DNA (later checked using the length histograms obtained after sequencing). We take care to compare samples with similar extents of digestion.

An alternative method for mapping nucleosomes is the chemical method [14,35]. It depends on the fact that the ser-47 residue of H4 is located near the nucleosomal dyad and can be mutated to cysteine. Nuclei are treated with a chemical modification agent which reacts with the Cys residue to generate hydroxyl radicals which in turn cleave proximal DNA. The result is a double-stranded DNA break very close to the dyad. Extensive digestion results in a ladder of DNA bands corresponding to dyad-to-dyad distances (not linker-to-linker as in MNase-seq). These DNA fragments are subjected to paired-end sequencing. The chemical method has several important advantages over MNase-seq—it gives the precise length of the linker DNA between neighbouring nucleosomes and more accurate nucleosome positions because length variability is limited, and it avoids the AT bias inherent in MNase-seq. It has two important disadvantages—the modification conditions are harsh and the H4-S47C mutation is required. The mutant is easy to make in yeast, but the large number of H4 genes in higher organisms precludes mutant construction and an siRNA approach must be used instead [36]. More recently, an alternative mutant for chemical mapping, H3-Q85C, has been used to deduce both dyad positions and linker lengths [37]. Currently, MNase-seq is much more commonly used than chemical mapping because it is easier and faster, but that may change in the future.

Recent advances in single-cell technology have made it possible to map nucleosomes from a single cell (scMNase-seq) and to gain further insight into chromatin heterogeneity at the cellular level [38]. However, there are two important caveats: (i) Many nucleosomes in each cell will be missing from the map because it is not possible to sequence the entire library. This means that an NDR cannot be reliably distinguished from a missing nucleosome. (ii) For diploid cells it is not usually possible to tell which chromosome copy each nucleosome came from. Another exciting technology making an entrance is long-read sequencing, which can be used to map nucleosome footprints on the same long DNA molecule, obtained using DNA methylases which can only methylate linker DNA [39].

Finally, it should be noted that yeast is an excellent model organism for chromatin studies because of its small genome and because mutants are simple to construct. The small genome (~12 Mb) makes a huge difference to the cost of sequencing. The mouse and human genomes are ~3000 Mb, therefore ~250 times more nucleosome sequences are required to achieve the same coverage as the yeast genome. About 10 million nucleosome sequences are sufficient for a high quality nucleosome map in yeast; ~2.5 billion nucleosome sequences are needed for the same coverage of the mouse or human genome. Most MNase-seq studies using human and mouse cells have far fewer nucleosome sequences and so the map is unreliable at the unique sequence level (but may suffice for global analyses). Biological replicate experiments are crucial for determining whether trends identified by bioinformatic analysis are reproducible; therefore replicate data should never be combined.

## 4. Nucleosome-Depleted Regions, Nucleosome Spacing and Nucleosome Phasing

**Figure 3:**
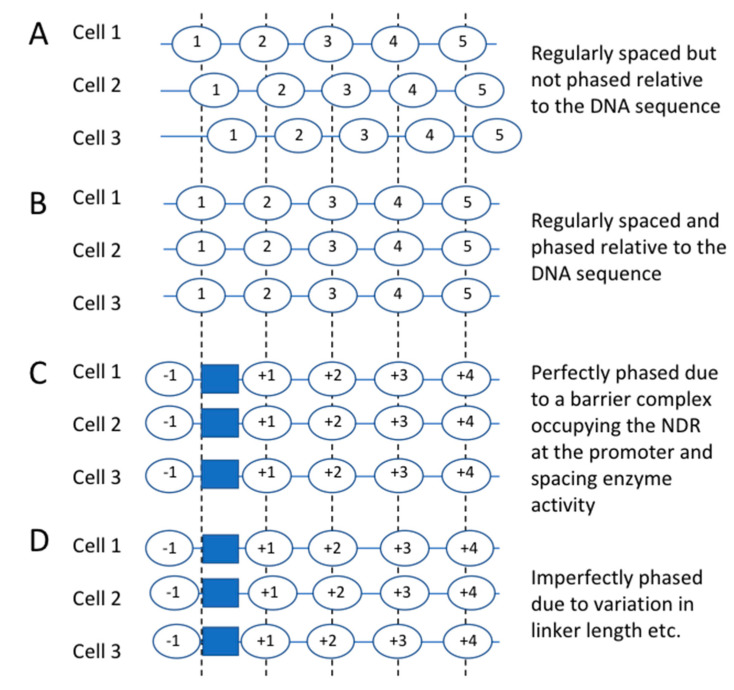
Nucleosome spacing and nucleosome phasing. (**A**) Nucleosomes can be regularly spaced but not phased on the same stretch of DNA. They are the same distance apart but in different positions in different cells. (**B**) Nucleosomes are regularly spaced and perfectly phased on the same stretch of DNA. They are the same distance apart and in the same positions in every cell. (**C**) Model for the origin of phasing. A putative “barrier complex” is formed at a specific sequence such as a promoter (blue box), preventing nucleosome formation on the DNA that it occupies and forcing nucleosomes to form on either side. If they are regularly spaced, phasing will result. (**D**) Model for phasing which takes into account observed variation in nucleosome positions and linker length (spacing).

Before we discuss the contributions of the various ATP-dependent remodelers to chromatin organisation, it is necessary to introduce the concept of nucleosome phasing. Nucleosome spacing refers to the average distance between nucleosomes, commonly measured by the repeat length in MNase studies (see above) (Figure 3A). Nucleosome phasing refers to the tendency of regularly-spaced nucleosomal arrays flanking active promoters, enhancers and other active regulatory elements to be phased relative to the underlying DNA sequence. That is, the nucleosomes tend to adopt similar positions at a given gene locus in all cells (Figure 3B).

Active regulatory elements are generally located in “nucleosome-depleted regions” (NDRs) or “nucleosome-free regions” (NFRs); we prefer to use “NDR” because these regions are usually not completely depleted of nucleosomes. In yeast, NDRs are present at most RNA polymerase II (Pol II) promoters [40,41], at genes transcribed by RNA polymerase III [42], and at replication origins. Phasing at promoters is revealed by aligning all ~5700 genes on their transcription start site (TSS) or on the average position of the first (“+1”) nucleosome position (dyad) on the gene and then mapping all of the nucleosome dyads to their gene coordinates. The result is a series of sharp peaks along the gene, corresponding to genomic average nucleosome positions relative to the TSS or +1 nucleosome (labelled +1, +2, +3 nucleosomes, etc.) [43,44]. In yeast, the TSS maps just inside the +1 nucleosome on average. Although the phased nucleosome peaks are strong, they are not infinite, and the troughs do not reach zero, indicating that phasing is very good but far from perfect. That is, nucleosomes are not precisely aligned and can be formed over linkers with lower probability. Single-gene studies have shown that nucleosomes adopt different overlapping positions on the same gene in different cells, both in yeast [45,46] and in higher organisms [47]. These alternative nucleosome positions account for imperfect phasing on the genomic scale, as shown by both MNase-seq and restriction enzyme accessibility data [48,49,50].

## 5. The NDR May Contain a “Barrier” Complex

The nature of the NDR is somewhat controversial. Weak NDRs can be formed in vitro using reconstituted nucleosomes and a combination of remodeling enzymes [51,52], indicating that DNA sequence plays an important role. Other studies have suggested the involvement of the runs of A-residues often present in yeast promoters, since they have a mild tendency to exclude nucleosomes. In vivo, promoter NDRs are deeper and wider. Interestingly, a peak is detected in NDRs at relatively early stages in MNase digestion, which disappears as digestion proceeds. This observation suggests that the NDR is not protein-free DNA, but occupied by a large stable complex [34,53,54,55,56,57]. It has been proposed that this complex is a “fragile nucleosome”, because it is more sensitive to digestion than a canonical nucleosome [55,56,57]. However, this is controversial. We were unable to detect any histones in these complexes, indicating that the MNase-sensitive complex is not a nucleosome, but a large non-histone complex [34]. We confirmed that this is true for the tRNA genes, which are marked by a deep NDR that is occupied by a stable, specifically bound transcription factor TFIIIB–TFIIIC(Transcription Factors IIIB and IIIC) complex with a footprint of ~150 bp, and flanked by phased nucleosomes [34,42].

At Pol II promoters, the sequence-specific general regulatory factors (GRFs: Abf1, Reb1 and Rap1) may be involved in NDR formation [58,59,60,61,62], either directly by acting as a barrier, or indirectly by recruiting a large stable transcription complex similar to the TFIIIB–TFIIIC complex. However, although depletion of these essential transcription factors reduces average NDR size, it does not eliminate the NDR, suggesting that either factor depletion is incomplete, or that more than one factor and/or other factors are involved in NDR formation [59,61]. We advocate for a model in which a specifically-bound, stable complex acts as a barrier, preventing nucleosome formation in the NDR, forcing nucleosomes to form on either side of the barrier, thus fixing their positions indirectly with respect to the DNA sequence (Figure 3C,D) [34,43,52,63,64]. A nucleosome-spacing enzyme would then build the phased array starting with the first (“+1”) nucleosome (see below).

## 6. The Yeast Nucleosome-Spacing Enzymes: ISW1a, ISW1b, ISW2, CHD1 and INO80

### 6.1. Nucleosome-Spacing Enzymes in Yeast Are Not Functionally Redundant

**Figure 4:**
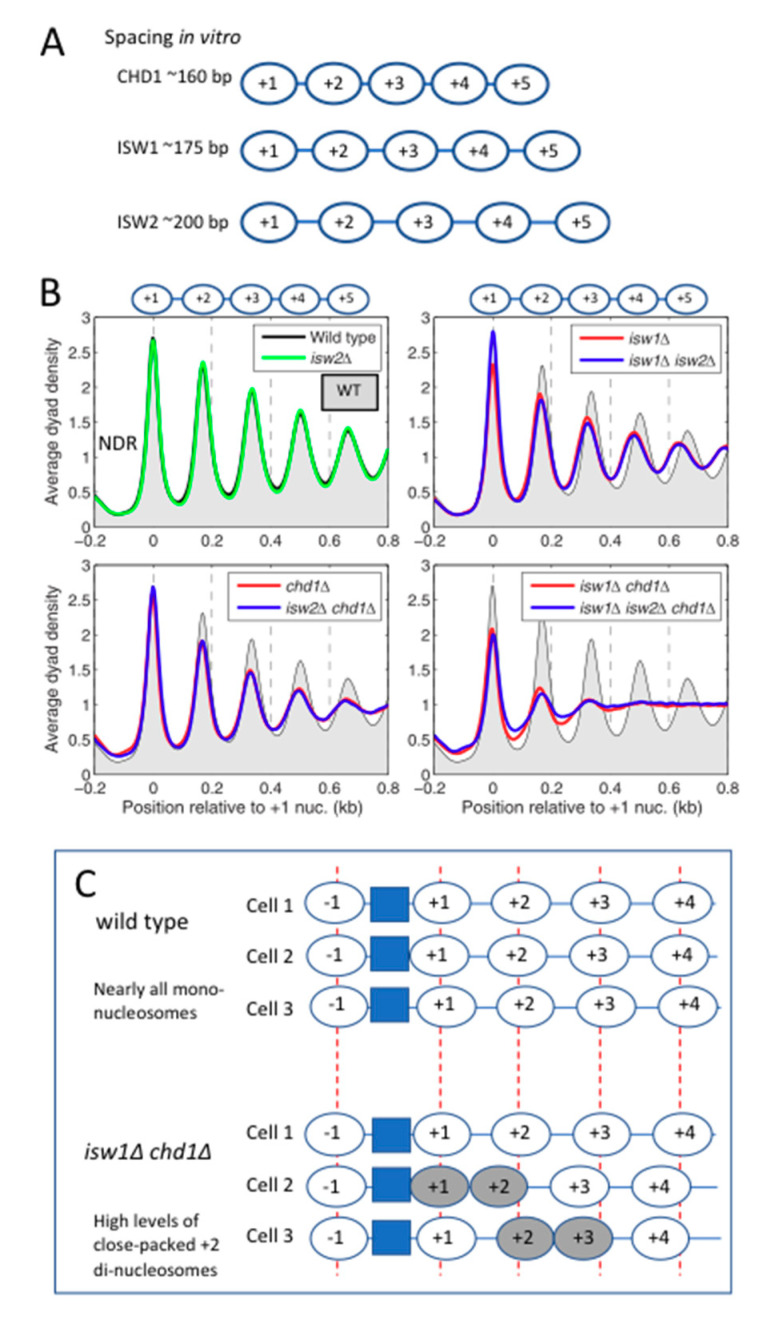
Yeast nucleosome-spacing enzymes in vitro and in vivo. (**A**) CHD1, ISW1 and ISW2 space nucleosomes differently in vitro. The nucleosomes are not phased. (**B**) The effects of null mutations in CHD1, ISW1 and ISW2 on chromatin organisation. Dyad phasing in wild type (WT) cells (black line with grey fill). All ~5700 yeast genes are aligned on the first (+1) nucleosome (set at 0). The genomic average dyad density is set at 1. The promoters are nucleosome-depleted regions (NDRs). Loss of ISW2 has no effect. Spacing is reduced in *isw1*Δ cells, while phasing is weaker in both *isw1*Δ and *chd1*Δ cells. Chromatin organisation in the *isw1*Δ *chd1*Δ double mutant is severely disrupted. A mathematical method to quantify the degree of phasing based on the relative height and width of each nucleosome peak is available [73]. Adapted from [73]. (**C**) Proposed contribution of close-packed di-nucleosomes involving the +1/+2 or +2/+3 nucleosomes (grey ovals) to phasing disruption in the *isw1*Δ *chd1*Δ double mutant. A di-nucleosome will alter the positions of downstream nucleosomes. In addition, the nucleosomes included in di-nucleosomes will be missing from this mono-nucleosome analysis, resulting in an apparent decrease in signal around the +2 nucleosome.

Yeast possesses five major chromatin remodelers with proven nucleosome-spacing activity: CHD1, ISW1a, ISW1b, ISW2 and INO80. In vitro, they form nucleosomal arrays with different average spacing. CHD1 establishes the shortest spacing (~160 bp), the ISW1 and INO80 complexes direct intermediate spacing (~175 bp), whereas ISW2 forms nucleosomal arrays with the longest spacing (~200 bp) [15,65,66,67,68,69,70,71,72] (Figure 4A). In yeast cells, the average spacing is ~165 bp, but there is significant variation, depending on the gene and its transcriptional activity (discussed below).

In vivo, normal nucleosome spacing and phasing relative to the +1 nucleosome requires primarily ISW1 and CHD1, while ISW2 makes only a marginal contribution at the global level (Figure 4B). The absence of ISW1 is the most detrimental for average nucleosome-spacing conservation. Early studies observed that, in *isw1*Δ cells, nucleosomes near the middle of a gene exhibit larger shifts towards the promoter than those closest to the promoter [74,75]. With the advent of paired-end technology, which allows greater certainty in the determination of the dyad location, it became clear that the differential nucleosome shifts that occur in the absence of ISW1 actually represent a decrease in the average nucleosome spacing, from ~166 bp in wild type cells to ~159 bp [73]. This observation implies that ISW1 creates nucleosomal arrays with wild type spacing. The phasing in *isw1*Δ cells is weaker than in wild type, as indicated by broader peaks with reduced amplitude. In the absence of CHD1 (*chd1*Δ), nucleosome spacing is slightly shorter than wild type and the phasing is weaker. Most importantly, the loss of both enzymes (the *isw1*Δ *chd1*Δ double mutant) has a much more drastic impact than either single mutant (Figure 4B), indicating that ISW1 and CHD1 cooperate to organise yeast chromatin [73,76].

The disruption of chromatin organisation in the *isw1*Δ *chd1*Δ double mutant involves an almost complete loss of downstream nucleosome phasing, beginning with the +2 nucleosome (Figure 4B). Remarkably, the +1 nucleosome remains well-positioned, due to the action of other remodelers (see below). More recently, we have identified a major contributory factor to the loss of phasing in the *isw1*Δ *chd1*Δ double mutant—the tendency to form close-packed di-nucleosomes [77]. These are neighbouring nucleosomes with no intervening linker DNA, which are therefore relatively resistant to MNase. Close-packed di-nucleosomes can be formed in vitro in the absence of remodelers by partial uncoiling of the DNA between two nucleosomes allowing close approach and apparent invasion and/or by elimination of an H2A–H2B dimer [78,79,80]. Di-nucleosomes can also be generated in vitro from mono-nucleosomes reconstituted on a plasmid by human SWI/SNF [81].

Surprisingly, the di-nucleosomes formed in the *isw1*Δ *chd1*Δ double mutant are not randomly located, but predominantly include the +2 nucleosome (i.e., the +1/+2 and +2/+3 di-nucleosomes are much more common than other possible di-nucleosomes) [77]. If we assume that downstream nucleosomes are regularly spaced relative to the di-nucleosome, then a major effect of di-nucleosome formation on phasing is predicted, because downstream nucleosomes are out of phase by one linker length (Figure 4C). Furthermore, since only mono-nucleosomes are included in the dyad plot, the formation of di-nucleosomes will result in a loss of dyad signal around the +2 nucleosome (Figure 4C). These effects can account for much of the chromatin disruption in the *isw1*Δ *chd1*Δ double mutant. Overall, the implication is that, in wild type cells, ISW1 and/or CHD1 resolve di-nucleosomes, or prevent their formation. Which of the two enzymes is involved, or whether both are required, is an outstanding question.

Loss of ISW2 (*isw2*Δ) does not impact global chromatin organisation [73,76]. In wild type cells, the role of ISW2 is masked by the dominant roles of ISW1 and CHD1; its contribution only becomes apparent in the *isw1*Δ *chd1*Δ *isw2*Δ triple mutant. In the *isw1*Δ *chd1*Δ double mutant, the global average spacing is difficult to measure accurately because the peaks are weak and flattened out. However, it can be estimated for the least transcriptionally-active genes. These genes show better phasing than the global average, with longer spacing than in wild type cells. The fact that this effect is abolished in the *isw1*Δ *chd1*Δ *isw2*Δ triple mutant suggests that ISW2 is responsible for most of the residual phasing and spacing in the *isw1*Δ *chd1*Δ double mutant [73]. Hence, ISW2 sets longer spacing and improves phasing on inactive genes, suggesting a role in repression. More direct evidence for ISW2 in a repressive role comes from studies showing that ISW2 can slide nucleosomes over promoters, coincident with repression [82] and that ISW2 represses cryptic transcript initiation (i.e., transcription from intragenic sites) [83].

In vitro, the INO80 complex displays nucleosome spacing and sliding activity [69] and can create NDRs with good positioning of the +1 and −1 nucleosomes in reconstituted chromatin [51]. In vivo, in the absence of INO80, the +1 nucleosome is shifted downstream, away from the TSS, whereas the downstream nucleosomes (+2, +3 etc.) are shifted upstream, towards the TSS [75,84], resulting in shorter average nucleosome spacing. Thus, INO80 is important for formation of the NDR and for spacing nucleosomes.

In summary, yeast nucleosome-spacing enzymes are not functionally redundant but instead direct the formation of nucleosome arrays with different spacing. Thus, the spacing on a particular gene depends on which of the three enzymes act on it. In general, CHD1 and ISW1 determine the spacing on most genes, whereas ISW2 affects only the least-active genes. The relative importance of INO80 has not yet been established.

### 6.2. ISW1 and CHD1 Compete to Set Nucleosome Spacing on Most Genes

As discussed above, ISW1 and CHD1 are the major spacing enzymes working at most genes. We have proposed that ISW1 and CHD1 compete to set the proper spacing [73]. Examination of the *isw1*Δ mutant illuminates the roles of both ISW1 and CHD1; the short nucleosome spacing in this mutant leads to two main conclusions: (1) the short spacing is set by CHD1, since it is the only other major spacing enzyme and (2) ISW1 is responsible for the longer spacing observed in wild type cells. If instead we focus on the *chd1*Δ mutant, the spacing is only slightly reduced, and we might be tempted to think that CHD1 has only a minor role. However, the importance of CHD1 is clear from the dramatic disruption of chromatin in the *isw1*Δ *chd1*Δ double mutant. An important issue is that the competition model predicts that the spacing in *chd1*Δ cells should be longer than in wild type, not slightly shorter, because the short-spacing enzyme is absent. A tentative explanation is that ISW1 function is partly dependent on CHD1, resulting in weakened phasing and spacing set by ISW1 in *chd1*Δ cells. That is, ISW1 may create more regular (better phased) arrays if the nucleosomes are first spaced by CHD1 and/or if di-nucleosomes are resolved by CHD1 (see above). We also note that the relative importance of the ISW1a and ISW1b complexes [65] to ISW1-dependent spacing is unknown.

In summary, we propose that ISW1 dominates the spacing on most genes, setting a longer spacing of ~165 bp, while CHD1 dominates genes with shorter spacing [73], which tend to be more active and may reflect association of CHD1 with Pol II elongation factors [85]. In addition, ISW2 makes a significant contribution to the longer spacing observed on the most inactive genes, where the competition may be between ISW1 and ISW2. Although in vitro studies indicated that CHD1, ISW1 and ISW2 should give spacings of ~160, ~175 and ~200 bp, only CHD1 gave the expected spacing in vivo (~159 bp). Nevertheless, the spacings deduced from the null mutant data in vivo are in the expected order (CHD1 < ISW1 < ISW2). In conclusion, the spacing enzymes compete to set the spacing on individual genes, and the global average spacing reflects the outcome of this competition [73].

### 6.3. Competing Remodelers May Control H1 Binding and Chromatin Folding by Regulating Spacing

Both the H3 N-terminal tail domain and yeast H1 (yH1) bind to the linker DNA, as shown by ChIP (chromatin immunoprecipitation) -exo experiments (such experiments markedly increase the resolution of standard ChIP experiments by using an exonuclease to digest the DNA up to the formaldehyde-crosslinked base) [86]. Using the same data, we showed that yH1 peaks are phased relative to the TSS but located over the linkers between nucleosomes, i.e., they are out of phase with the nucleosome peaks [73]. Moreover, yH1 binding is detected on the linkers of all genes except for those with extremely short or extremely long spacing, both of which are indicative of heavy transcription (see below). Hence, genes with short spacing, determined primarily by CHD1, bind less H1 than genes with longer spacing, determined primarily by ISW1 [73].

yH1 differs from mammalian H1 in that it has two globular domains instead of one; they are separated by a stretch of ~40 residues that is homologous to the highly-positively-charged C-terminal tail domain of mammalian H1 [87]. The globular domain of mammalian H1 interacts with the nucleosome core, while the C-terminal tail follows the linker (reviewed in [88]). The unusual structure of yH1 suggests that it has the potential to interact with two nucleosome cores and with the intervening linker DNA [89,90,91]. Deletion of the gene encoding yH1 (*HHO1*) does not affect nucleosome spacing in vivo [89,92,93]. This may reflect the much lower stoichiometry of H1 per nucleosome in yeast (1 per ~37 nucleosomes [94]) than in higher organisms (~1 per nucleosome [95]). If yH1 levels are low in wild type cells, loss of yH1 would affect the spacing of just a small fraction of nucleosomes, which may not be apparent at the global level.

**Figure 5:**
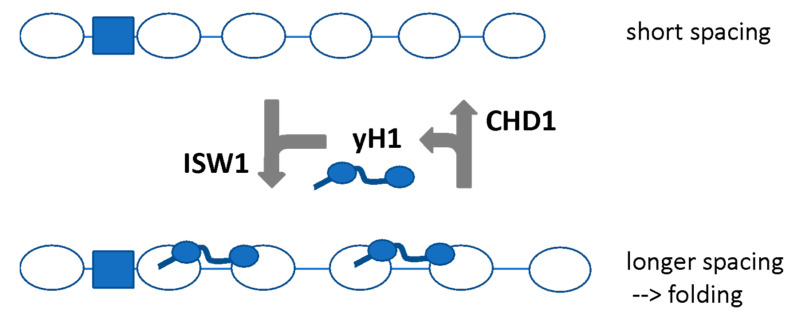
Model proposing that ISW1 and CHD1 regulate chromatin folding by determining nucleosome spacing. ISW1 increases the spacing resulting in longer linkers which can bind yH1 with high affinity and condense the chromatin. CHD1 reduces the spacing, resulting in eviction of yH1 and unfolding. Adapted from [73].

We proposed that CHD1 directs short spacing, evicting H1 because the linker is too short for high-affinity binding, which in turn results in partial unfolding of the chromatin fibre. In contrast, ISW1 directs longer spacing, facilitating the binding of yH1 by creating a longer linker, which results in re-folding of the chromatin (Figure 5). Thus, a dynamic competition between ISW1 and CHD1 may control chromatin folding by regulating yH1 binding [73]. Since loss of yH1 has no clear phenotype and only minor effects on gene expression [89,94], we propose that the primary function of yH1 is to condense genic chromatin during periods of inactivity.

### 6.4. Nucleosome-Spacing Enzymes and Transcription

Given that ISW1 and CHD1 have such a profound effect on global chromatin organisation, it is surprising that neither the *ISW1* gene nor the *CHD1* gene is essential and that both genes can be deleted without resulting in a strong phenotype. This is also true of ISW2 and the triple mutant. This observation indicates that highly-organised chromatin is not critical for essential functions. Consistently, the loss of these remodelers is accompanied by relatively minor changes in gene expression, as measured by ChIP-seq for the Rpb3 subunit of Pol II [73] or by RNA microarray [76]. Isw1 is involved in the repression of certain stress response genes during normal growth [96].

**Figure 6:**
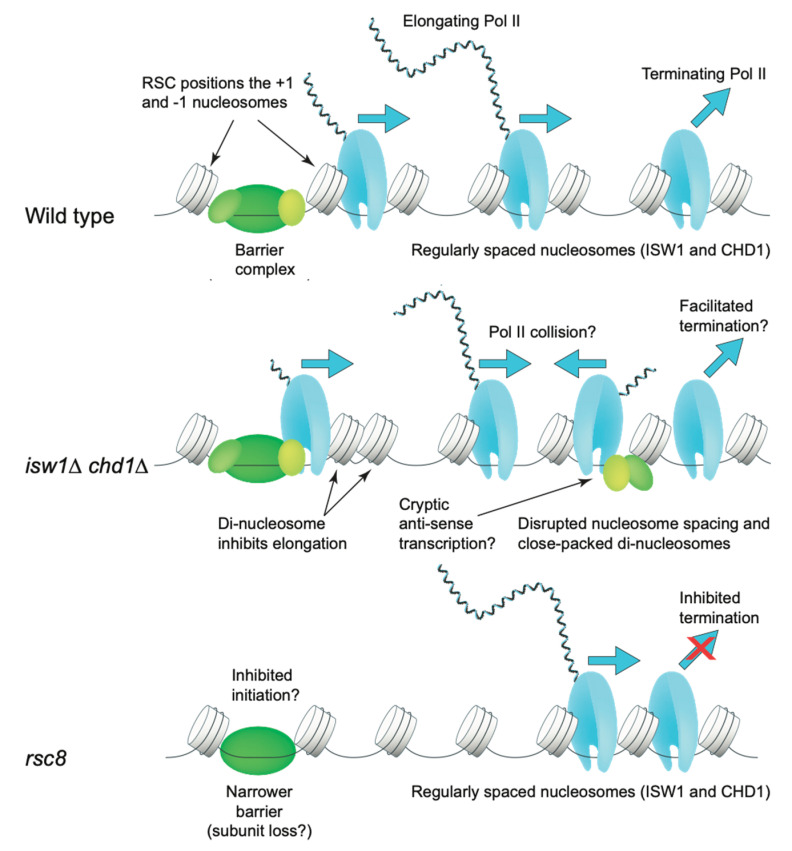
ATP-dependent remodelers and transcription. In wild type cells, Pol II levels are low at the promoter (promoter–proximal pausing is not generally observed in yeast), high on the gene body (elongating Pol II), and there is a peak of terminating Pol II downstream of the transcript termination site (TTS). In the *isw1*Δ *chd1*Δ double mutant, elongating Pol II levels are high relative to terminating Pol II levels, suggesting that elongation is inhibited and/or termination is facilitated. In the former case, close-packed di-nucleosomes may inhibit elongation; in addition, increased anti-sense cryptic transcription may result in inhibition due to Pol II collisions. In Rsc8-depleted cells, terminating Pol II levels are high relative to elongating Pol II, suggesting that termination is inhibited. Transcription levels are very low in these cells, suggesting an initiation defect also. Adapted from [77].

Interestingly, the distribution of Pol II on the average gene body is affected in *isw1*Δ *chd1*Δ cells. In wild type cells, Pol II levels are low at the promoter and high on the gene (elongating Pol II), with a dip at the transcript termination site (TTS), and a peak just downstream from the TTS that sometimes trails into the next gene, corresponding to terminating Pol II [97,98]. The TTS corresponds to the nucleotide at which the mRNA is cleaved and tailed with poly(A); transcription continues for a short distance downstream of the TTS. In the *isw1*Δ *chd1*Δ double mutant, the peak representing terminating Pol II is significantly decreased [77]. This effect is more pronounced in *chd1*Δ cells than in *isw1*Δ cells. Thus, ISWI and CHD1 may delay termination and/or dissociation of Pol II. Alternatively, ISW1 and CHD1 may facilitate Pol II elongation, such that elongation is inhibited in *isw1*Δ *chd1*Δ cells, possibly by close-packed di-nucleosomes, resulting in higher levels of Pol II on the gene relative to the termination region [77]. Another factor is cryptic initiation, which is increased in the *isw1*Δ *chd1*Δ double mutant [99], such that elongation may also be inhibited by collisions between promoter-initiated Pol II and cryptically-initiated Pol II elongating in the anti-sense direction (Figure 6).

Transcription generally has quite minor effects on chromatin structure, unless the gene is heavily transcribed [97,100,101]. This observation may indicate that the chromatin organisation of genes that are transcribed less often is restored by remodelers once transcription is complete, whereas the chromatin structures of genes with high levels of Pol II are not restored quickly enough. In yeast, only the ~50 most-heavily-transcribed genes (as measured by ChIP-seq for the Rpb3 subunit of Pol II) show obvious chromatin disruption [48,97]. Heavily-transcribed genes are associated with a wider NDR, decreased nucleosome occupancy, disrupted phasing on gene bodies and short spacing [48,53,97,102,103,104,105]. Loss of occupancy reflects some transcription-associated loss of nucleosomes and, more commonly, loss of H2A–H2B dimers from existing nucleosomes, resulting in sub-nucleosomes [63,86,97].

The short spacing on heavily-transcribed genes is puzzling, because it implies that highly-active genes have more nucleosomes, which is apparently inconsistent with nucleosome loss. That there is a link between spacing and transcription is supported by the global long spacing observed in the complete absence of transcription using the temperature-sensitive Pol II *rpb1–1* mutant [53]. These data may be reconciled by arguing that the short spacing on heavily-transcribed genes reflects a general disruption due to transcription, such that small clusters of closely-spaced nucleosomes are formed, perhaps as Pol II transcribed through them, separated by occasional gaps (long linkers) resulting from nucleosome loss or hexasome formation (i.e., loss of an H2A–H2B dimer). The short spacing may be directed by CHD1, since there is evidence that CHD1 is associated with Pol II elongation factors [85], implying that genes with more Pol II have more CHD1. Intriguingly, it may be relevant that Chd1 can slide hexasomes unidirectionally in vitro [106]. We have also observed that some heavily-transcribed genes have extremely long spacing [73], although this was not observed in chemical mapping experiments for reasons that are not clear [37]. Extremely long spacing could be explained by relatively high nucleosome or H2A–H2B dimer loss and/or by reduced levels of CHD1.

Single-gene studies suggest a more central involvement of nucleosome-spacing enzymes in coordinating transcriptional events. Two nucleosomes reminiscent of a di-nucleosome are formed at the 5′-end of the *MET16* gene in an *isw1*Δ mutant [107]. It was proposed that the ISW1a complex positions nucleosomes at the promoter to regulate initiation, whereas ISW1b acts in the coding region to facilitate Pol II elongation and termination [107,108]. In support, ChIP-exo studies show that ISW1a is enriched at gene-terminal nucleosomes, whereas ISW1b is enriched on gene bodies [75]. CHD1 has also been implicated as a transcription termination factor in a single-gene study [108]. However, critical global roles in transcription for the ISW1 and CHD1 complexes seem unlikely in view of the weak phenotypes of the null mutants.

## 7. Nucleosome Remodeling Enzymes: RSC and SWI/SNF

### 7.1. Remodeling Activities of the Yeast SWI/SNF-Family Complexes, RSC and SWI/SNF

The SWI/SNF and RSC complexes have similar remodeling activities in vitro. They are both capable of translocating nucleosomes (i.e., sliding the histone octamer along the DNA or transferring it to another DNA molecule) by expending ATP [17,109,110,111]. Although they can slide nucleosomes, they are not able to organise nucleosomes into regularly-spaced arrays. They can both convert canonical nucleosomes into alternative conformational states—SWI/SNF can generate “altosomes” by forcing the coalescence of mono-nucleosomes into di-nucleosomes [81] and then disassemble one of the nucleosomes [79] and RSC can unravel nucleosomes [112] and form “remosomes” by converting nucleosomes to a much more open conformation incorporating more DNA (~180 bp) [113,114]. Such remodeled nucleosomes could be important for regulating transcription, but it is unclear whether altosomes or remosomes occur in vivo. In vitro studies designed to reconstitute native yeast chromatin organisation using purified histones, DNA and remodelers (i.e., to create an NDR and phased nucleosomes with appropriate spacing) reveal that RSC and INO80 can separately create NDRs at promoters [51].

### 7.2. Roles of RSC and SWI/SNF in Determining NDRs In Vivo

**Figure 7:**
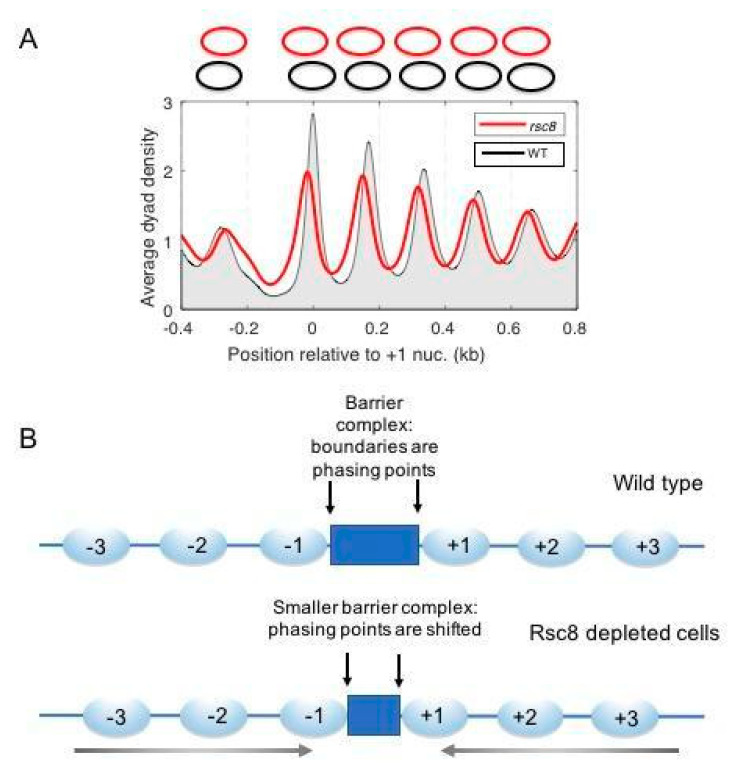
Contribution of the essential remodeling the structure of chromatin (RSC) complex to yeast chromatin organisation. (**A**) Loss of RSC results in nucleosome shifts into the promoter, narrowing and partially filling the NDR. Nucleosome spacing is unchanged, indicating that all of the nucleosomes shift towards the promoter. Cells were depleted of the Rsc8 subunit by repressing its expression from a *GAL* promoter. (**B**) The effect of RSC depletion can be explained by a smaller putative barrier complex footprint, perhaps involving a loss of barrier subunits. Adapted from [77].

Although the RSC and SWI/SNF complexes have similar remodeling activities in vitro, their functions appear to be largely non-redundant in vivo. Indeed, RSC is essential [115], whereas SWI/SNF is not (although *snf2*Δ cells grow very slowly, indicating that it fulfills important functions). RSC plays an important role in NDR formation, as shown by the narrowing and moderate filling in of the NDR when essential RSC subunits are depleted from cells [59,77,116,117] (Figure 7A). However, deep NDRs are still present even in the absence of RSC. Therefore NDR formation is only partly dependent on RSC unless depletion of one subunit is not sufficient to fully inactivate RSC. In contrast, SWI/SNF has little effect on the NDR at the global level, since cells lacking the crucial ATPase subunit (*snf2*Δ) do not display a global defect in chromatin organisation [116].

In essence, NDR formation involves the positioning of the +1 and −1 nucleosomes with a gap in between. Several remodelers appear to play a role in positioning the +1 and −1 nucleosomes in vivo. RSC has the most obvious effect on the +1 and −1 nucleosomes since RSC depletion results in an inward shift of both nucleosomes at most promoters. This effect is partly reversed by ISW1 [77,118]. Similarly, at inactive genes, ISW2 shifts the +1 nucleosome towards the promoter, in the opposite direction to RSC [73,83]. Loss of INO80 results in +1 nucleosome shifts in the opposite direction to RSC, effectively widening the NDR [75,84]. Although loss of SWI/SNF does not affect global +1 and −1 nucleosome positioning, it does reduce the depth and width of the NDRs at the promoters of highly-expressed genes, where it cooperates with other factors including RSC and INO80, the Gcn5 histone acetyltransferase and the histone chaperone Ydj1 [28,117,119]. Overall, though, remodeler-dependent changes in +1 and −1 nucleosome positions and hence NDR width are generally small, averaging no more than ~20 bp. A small shift in position could expose or bury specific binding sites or the TSS in a nucleosome, suggesting a mechanism for gene regulation. However, the heterogeneity in +1 nucleosome positions in the cell population renders this type of model unsatisfying, because a particular specific site will be exposed in some cells, but not in others, predicting that only some cells can respond [49].

If RSC acts directly on the +1 and −1 nucleosomes, RSC would be expected to be concentrated at its target promoters. Surprisingly, this is not at all clear. MNase-ChIP-seq experiments (in which chromatin from formaldehyde crosslinked cells is digested with MNase to release mono-nucleosomes for immunoprecipitation) show that RSC is bound to the −1 and +1 nucleosomes, which is as expected, but also to the +2 and +3 nucleosomes, suggesting that RSC may also act on nucleosomes on gene bodies [75]. Native ChIP experiments (similar to MNase-ChIP-seq but without crosslinking) are quite consistent, indicating that RSC preferentially binds to the +1 and −1 nucleosomes [120]. “Cut-and-run” experiments, in which a Protein A-MNase fusion protein is bound to the RSC ATPase subunit (Sth1) in nuclei via an antibody bridge, also suggest that RSC binds preferentially to the +1 and −1 nucleosomes [57]. This study also proposes that RSC binds to a remodeled sub-nucleosome in promoters with wide NDRs [57], although the possibility that free Protein A-MNase (not bound to Sth1) might digest chromatin independently of Sth1 binding was not addressed. In contrast, standard ChIP experiments (which do not involve MNase and rely on formaldehyde to crosslink the target protein to DNA before the cells are disrupted) indicate that RSC is not enriched at promoters, but modestly enriched on active genes [116,117,121,122,123], suggesting that RSC remodels genic chromatin rather than promoter chromatin.

There is a similar difficulty with data for SWI/SNF—MNase-ChIP-seq experiments indicate that SWI/SNF is bound to the +1 and −2 nucleosomes [75], whereas ChIP experiments indicate that it is modestly enriched on active genes [117]. There are also a number of single-gene studies showing activator-dependent enrichment of SWI/SNF at specific promoters and genes, supporting a transcription factor recruitment mechanism (e.g., [124,125,126]. In addition, there is evidence for activator-dependent, SWI/SNF-dependent chromatin disruption over the entire *HIS3* gene [46,127]. Clearly, there are contradictions in the literature concerning the genomic distributions of SWI/SNF and RSC that need to be resolved if we are going to understand their functions in detail. The answer may lie in the fact that the RSC and SWI/SNF enrichments reported by the various methods are all rather small and perhaps over-emphasised.

We have suggested the possibility of an indirect role for RSC in NDR formation—that the changes in NDR width actually reflect changes in the footprint of the putative barrier complex that resides in the NDR [34,77]. In this model, the positions of the +1 and −1 nucleosomes are not dictated directly by remodelers, but by the size of the barrier complex (Figure 7B). The putative transcription complex in promoter NDRs may lose subunits, given that transcription is severely curtailed in RSC-depleted cells [128]. Our model is not completely consistent with the in vitro data indicating that RSC and INO80 can create an NDR in the absence of a barrier complex [51], although the NDR formed in vitro is shallow and is partly attributable to DNA sequence effects [52]. The model does account for the genic distribution of RSC in vivo, suggesting that RSC really functions on genes during transcription. Clearly, more experiments are needed to resolve this critical issue.

### 7.3. RSC, SWI/SNF and Transcription

As discussed above, the distribution of Pol II on the average gene includes a peak of terminating Pol II just downstream of the TTS. In the absence of RSC (cells depleted of the essential Rsc8 subunit by repressing *RSC8* expression from a *GAL* promoter), the amount of terminating Pol II is increased relative to elongating Pol II, implying a role for the RSC complex in facilitating transcript termination [77]. The accumulation of terminating Pol II in *rsc8* cells suggests that termination is inhibited, which might explain the generally low levels of mRNA in RSC-depleted cells [128], although this could also be accounted for by an initiation defect. In wild type cells, it has been proposed that Pol II termination or dissociation might be slow in order to facilitate recycling of Pol II from just downstream of the TTS back to the promoter without release into the nucleoplasm [97,129]. If RSC is involved in initiation, Pol II recycling might be prevented, resulting in Pol II accumulation downstream of the TTS (Figure 6). An effect of RSC on initiation is also suggested by data indicating that the extent of repression of genes in the absence of RSC depends on whether all of the alternate TSSs, or only some of them, are occluded by the +1 nucleosome when it shifts into the promoter [130]. RSC might be directly involved in initiation through its role in NDR formation, or indirectly through partial disassembly of the barrier complex, as discussed above [34,116,117].

Nucleosome mapping, Pol II ChIP-seq and gene expression data all indicate that RSC affects many more genes than SWI/SNF [77,116,117,119,131]. Both SWI/SNF and INO80 stimulate transcription specifically at highly-expressed genes, where it augments RSC function [28,117,119]. Another role for SWI/SNF in transcription is suggested by somewhat controversial evidence that SWI/SNF binds directly to Pol II and so might facilitate Pol II binding to promoters and/or facilitate transcription through nucleosomes [132].

### 7.4. Dynamic Nucleosome Remodeling by RSC In Vivo

Both MNase-seq and chemical mapping of nucleosomes require the preparation of nuclei (crude or otherwise). Consequently, the chromatin structure is probably “frozen” because remodelers are unable to function without ATP. Thus, the dynamic aspects of chromatin structure can only be inferred from the cell population average. Study of the dynamic events involved in transcription in live cells therefore requires different approaches. Biophysical experiments show that the binding of transcription factors to their specific sites is rapid and reversible on the time-scale of several seconds [133]. The binding and dissociation of transcription factors is thought to be regulated by ATP-dependent chromatin remodelers [134] which may control site access by moving nucleosomes on or off specific binding sites, since nucleosomes inhibit binding. The single molecule tracking method has provided insight into transcription factor and remodeler dynamics [135,136,137,138,139]. In yeast, single molecule tracking has been used to address the role of RSC in the activation of the *CUP1* gene by the Ace1 transcription factor, which binds to specific sites in the *CUP1* promoter [140]. This study shows that RSC improves the search and binding kinetics of Ace1 to the promoter, probably through its fast nucleosome remodeling activity (in the range of a few seconds), removing promoter nucleosomes or shunting them back and forth, alternately exposing and burying Ace1 binding sites, and leading to rapid bursts of *CUP1* transcription. New methods to map nucleosomes in living cells may shed more light on chromatin dynamics.

## 8. Important Issues for Future Study

There are a number of important unresolved issues concerning the roles of the ATP-dependent remodelers in yeast chromatin organisation. They include reaching a better understanding of the contribution of INO80 to global nucleosome spacing and dissecting the individual contributions of the two ISW1 complexes (ISW1a and ISW1b). In addition, the respective contributions of ISW1 and CHD1 to prevention of di-nucleosome formation need to be determined. Furthermore, the significance of di-nucleosome formation in the context of transcription is unclear. Our model proposing that ISW1 and CHD1 indirectly determine chromatin compaction by determining spacing and H1 binding requires further testing. A closer examination of the transcripts produced in the various remodeler mutants is warranted, particularly in view of evidence indicating that ISW1 interacts with mRNP(mRNA nucleoproteins) in the nucleus, regulating mRNP export [141]—are the transcripts correctly initiated and terminated? Finally, as discussed above, it is essential to resolve the issue of the genomic locations of SWI/SNF and RSC to reach an understanding of their functions.

## 9. Conclusions

In budding yeast, chromatin organisation is determined by the interplay of several ATP-dependent chromatin remodeling complexes. We expect the same is true of higher eukaryotes, which have many more ATP-dependent chromatin remodelers than yeast. Their mechanisms of action have been studied in depth in vitro and elucidated further by recent high-resolution cryo-electron microscopy studies. With the advent of next generation sequencing, their roles in vivo are now much better understood. However, there are still unresolved issues that require further research.
